# Prenatal diagnosis and family analysis of 17q12 microdeletion syndrome with fetal renal abnormalities

**DOI:** 10.3389/fgene.2024.1401315

**Published:** 2024-06-18

**Authors:** Fang Zhang, Qingqing Gu, Jiedong Song, Yali Zhao, Zhiwei Wang, Shuai Men, Leilei Wang

**Affiliations:** Department of Prenatal Diagnosis, Lianyungang Maternal and Child Health Hospital, Lianyungang, China

**Keywords:** 17ql2 microdeletion syndrome, HNF1B, prenatal diagnosis, unreported expressions, family analysis

## Abstract

**Purpose:**

To analyze the prenatal diagnosis, parental verification, and pregnancy outcomes of three fetuses with 17ql2 microdeletion syndrome.

**Methods:**

We retrospectively reviewed 46 singleton pregnancies with anomalies in the urinary system who underwent amniocentesis from Feb 2022 to October 2023 in the Prenatal Diagnosis Center of Lianyungang Maternal and Child Health Hospital. These fetuses were subjected to chromosomal microarray analysis (CMA) and/or trio whole-exome sequencing (Trio-WES). We specifically evaluated these cases’ prenatal renal ultrasound findings and clinical characteristics of the affected parents.

**Results:**

Three fetuses were diagnosed as 17q12 microdeletions, and the detection rate was 6.5% in fetuses with anomalies in the urinary system (3/46). The heterogeneous deletions range from 1.494 to 1.66 Mb encompassing the complete hepatocyte nuclear factor 1 homeobox B (*HNF1B*) gene. Fetuses with 17q12 deletion exhibited varied renal phenotypes. Moreover, the clinical phenotypes of the affected parents differed greatly in the two cases (case 2 and case 3) in which the deletion was inherited. For case 3, the mother manifested classic symptoms of 17q12 deletion syndrome as well as unreported characteristics, such as very high myopia.

**Conclusion:**

Our findings demonstrate the necessity and significance of offering prenatal genetic testing when various renal anomalies are detected. In addition, our study broadens the phenotypic spectrum of 17q12 deletions. Most importantly, our findings may allow timely supportive genetic counseling and guidance for pregnancy in affected families, e.g., with the help of preimplantation genetic testing (PGT).

## Introduction

The 17q12 microdeletion syndrome is a recurrent copy number variant (CNV) characterized by a 1.06–2.46 Mb deletion that encompasses the hepatocyte nuclear factor 1 homeobox B (*HNF1B*) [OMIM 189907] gene (Moreno-De-Luca et al., 2010; Wu et al., 2021). The reported prevalence of this microdeletion syndrome is between 1:14,000 and 1:50,000, with high penetrance and variable expressivity (Rosenfeld et al., 2013; Martin et al., 2020). Unlike most genomic disorders, the 17q12 deletion was once thought to be one of the rare genetic abnormalities devoid of any developmental delay or intellectual disabilities (Mefford et al., 2007). However, more recent studies have described patients with this pathogenic CNV who were referred for genetic testing because of neurodevelopmental abnormalities, including autism spectrum disorders (ASD) and schizophrenia, rather than renal abnormalities (Moreno-De-Luca et al., 2010; Palumbo et al., 2014; Laffargue et al., 2015). Congenital diaphragmatic hernia, hypotonia, seizures, and genitourinary defects, including Mayer-Rokitansky-Kuster-Hauser syndrome, duodenal atresia, and newborn cholestasis were also reported seldom in patients with 17q12 microdeletions (Bernardini et al., 2009; Yap et al., 2015; Su et al., 2022).

Due to the limitations of prenatal ultrasound, only obvious structural abnormalities can be detected prenatally, most commonly with renal involvement. Bilateral hyperechogenic kidneys with normal or slightly increased size can be detected by prenatal ultrasonography. Otherwise, neuropsychiatric disorders can be observed only after birth (Decramer et al., 2007). It is estimated that 20% of birth abnormalities are identifiable by ultrasound as fetal renal anomalies (FRA) (Dias et al., 2014), and congenital anomalies of the kidney and urinary tract (CAKUTs) are common birth defects in newborns. Due to the differences in sociodemographic background, malformation inclusion criteria, study population, diagnostic technology, and surveillance quality, the prevalence of CAKUTs varies greatly, ranging from 4.2 per 10,000 births in China to 4.0 per 1,000 births in European countries (Andrés-Jensen et al., 2016; Laurichesse Delmas et al., 2017). The large difference may be due to the differential inclusion criteria applied by the studies. While the former was restricted to only cases found in the first 7 days following delivery, the latter covered cases up to 22 weeks of gestation as well as infants up to 8 years old. Although fetal anomalies, such as renal pelvis dilatation and hyperechogenicity can occasionally resolve spontaneously with fetal maturation, a genetic condition such as 17q12 microdeletion syndrome may be an underlying genomic cause of these conditions (Verbitsky et al., 2019). Chromosomal microarray analysis (CMA) has been proven to be an efficient first-line diagnostic technique for fetal kidney anomalies, while trio whole-exome sequencing (Trio-WES) is a first-line test in patients exhibiting congenital anomalies (CA), developmental delay (DD), and intellectual disability (ID) (Groopman et al., 2018; Manickam et al., 2021).

With increased knowledge about prenatal diagnosis and advancements in molecular detection techniques, the prenatal diagnosis of 17q12 deletion syndrome has become more common. In this study, we retrospectively analyzed the clinical indications, ultrasonic manifestations, and molecular detection results of three fetuses with confirmed 17q12 deletion as well as their parents to expand the knowledge about the prenatal diagnosis of this syndrome.

## Materials and methods

### Ethics statements

This study was approved by the Ethics Committee of Lianyungang Maternal and Child Health Hospital (Number: LYG-MER2021038). Written informed consent for genetic testing and publication of the results was obtained from all the patients.

### Prenatal renal sonographic studies

The ultrasound screening was routinely performed for pregnant women by senior sonographers using GE E8 ultrasound machines (General Electric Healthcare, US) with a trans-abnormal curvilinear transducer of 4–8 MHz at the Lianyungang Maternal and Child Health Hospital.

## Patients and samples

A total of 46 singleton pregnancies with anomalies in the urinary system underwent amniocentesis from Feb 2022 to October 2023 in the Prenatal Diagnosis Center of Lianyungang Maternal and Child Health Hospital. These fetuses were subjected to CMA and/or Trio-WES. These families were all unrelated, healthy, non-consanguineous, and had no family history of genetic disease or congenital malformations. Information of three cases with confirmed 17q12 deletion was retrospectively collected, including the clinical characteristics, diagnostic procedures, diagnosis outcomes, and pregnancy outcomes and their family analysis.

### Quantitative fluorescent polymerase chain reaction

Genomic DNA was extracted from uncultured amniotic fluid using a QIAGEN kit (Qiagen, Hilden, Germany), according to the manufacturer’s instructions. To detect maternal cell contamination and polyploidy, quantitative fluorescent polymerase chain reaction (QF-PCR) was performed using a set of STR markers on chromosomes 13, 18, 21, X, and Y.

### Chromosomal microarray analysis

Genomic DNA was extracted from uncultured amniotic fluid using a QIAGEN kit (Qiagen, Hilden, Germany), according to the manufacturer’s instructions. Affymetrix CytoScan 750K (Affymetrix, Santa Clara, CA, USA) was used for chromosomal aneuploidy analysis. DNA digestion, amplification, segmentation, labeling, and hybridization of the arrays were performed according to the manufacturer’s instructions (Affymetrix, USA). The results were analyzed using the Chromosome Analysis Suite software (ChAS). The following public databases were used for data analysis and interpretation: Database of genomic variants (DGV; http://projects.tcag.ca/variation/), database of Chromosomal Imbalances and Phenotypes in humans using Ensembl Resources (DECIPHER; http://decipher.sanger.ac.uk/), online mendelian inheritance in man (OMIM; http://www.omim.org), ISCA search (http://dbsearch.clinicalgenome.org/search/), ClinVar of NCBI (https://www.ncbi.nlm.nih.gov/clinvar/) and ClinGen Dosage Sensitivity Map (https://dosage.clinicalgenome.org/).

### Whole-exome sequencing

Genomic DNA extracted from amniotic fluid and peripheral blood samples of the first and third pregnant woman and their husbands was sent for whole exome capture using the universal Kit for sequencing reaction (BGI, Shenzhen, China). According to the manufacturer’s recommendations, the resulting libraries were sequenced on a BGISEQ-500 platform (BGI, Shenzhen, China). Reads were aligned to the human reference genome (GRCh37/hg19) using a Burrows-Wheeler Aligner (BWA V0.7.15) (Li and Durbin, 2010; Lv et al., 2018). All genomic variations, including single-nucleotide polymorphisms (SNPs) and insertions/deletions (InDels) were detected and filtered by GATK HaplotypeCaller (v3.3.0) (Broad Institute, Cambridge, MA, USA (McKenna et al., 2010). Potential disease-causing mutations were predicted using the sorting intolerant from tolerant (SIFT) algorithm (Ng and Henikoff, 2003). Data were filtered with several variant databases, including dbSNP (https://www.ncbi.nlm.nih.gov/projects/SNP/), the 1000 Genomes Project (ftp://ftp-trace.ncbi.nih.gov/1000genomes/ftp/release), and the NHLBI-ESP6500 database (http://evs.gs.washington.edu/EVS/). Candidate mutations were expected to be absent from these databases. The conservation analysis of amino acid sequences was aligned using ClustalW2 (http://www.ebi.ac.uk/Tools/msa/clustalw2/).

### Genetic counseling

For those with 17q12 deletion syndrome, genetic counseling is a crucial procedure. Detailed genetic counseling was offered by trained genetics to all couples in our center. Prospective parents were educated about the potential phenotypes in addition to the reported incomplete penetrance and variable expressivity. The assessment of recurrent risk was also conducted based on the inheritance or *de novo* of the CNV. Treatment of manifestations in individuals with this deletion syndrome is symptomatic and depends on an individual’s specific needs. Once the 17q12 recurrent deletion has been identified in an affected family member, it is necessary that prenatal testing (through chorionic villus sampling and/or amniocentesis) for a pregnancy at increased risk, and preimplantation genetic testing is possible.

## Results

Of 46 fetuses, 33 (71.7%) had isolated urinary system anomalies, and 13 (28.3%) had non-isolated system anomalies. The whole frequency of chromosomal aberrations in fetuses with urinary system anomalies was 15.2% (7/46), including one case (2.2%) with trisomy 13, three cases (6.5%) with 17q12 deletion, and three cases (6.5%) with other likely pathogenic CNVs. 17q12 recurrent deletion syndrome was the most frequently detected microdeletion syndrome in our study (3/46, 6.5%). The general characteristics regarding prenatal sonographic findings, molecular analyses, and pregnancy outcomes of three cases with 17q12 deletion are presented in Table 1.

**TABLE 1:** General and genetic testing information of the three fetuses of 17 q12 deletion syndrome.

	Maternal age (y)	Gestational age at diagnosis (w)	Specimen	Prenatal ultrasonic findings	Testing results of the fetuses		Origin	Pregnancy outcome
					Karyotype	SNY array/Whole exome sequencing		
Fetus 1	21	23	AF	left polycystic kidney and decreased amniotic fluid amount	—	seq[GRCh37] 17q12(34,686,312_36349404)x1	*De novo*	TOP
Fetus 2	30	24 + 4	AF	left kidney dysplasia	—	arr[hg19]17q12(34,822,465-36410720)x1	Paternally	TOP
Fetus 3	25	18 + 3	AF	Smaller kidney sizes and bilateral hyperechogenic kidneys	46, XY	arr[hg19]17q12(34,822,466-36,316,144)x1	Maternally	TOP

Abbreviations: y, year; w, week; AF, amniotic fluid; TOP, termination of pregnancy.

### Case 1

**FIGURE 1:**
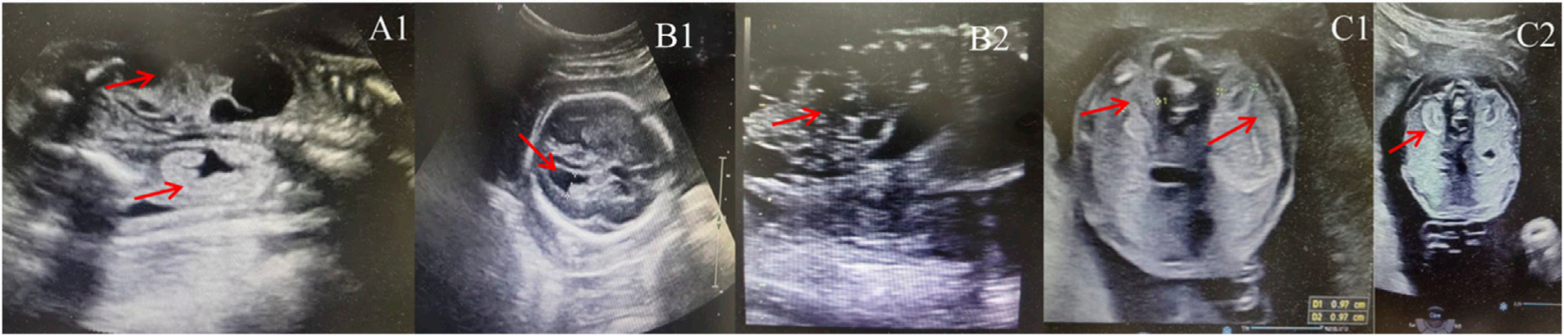
Sonographic characteristics of renal anomalies in fetuses with 17q12 deletion syndrome. **(A1)**: left multicystic dysplastic kidney and right hyperechogenic kidney of fetus case 1; **(B1)**: increased width of the posterior corner of the lateral ventricle of fetus 2 (10 mm); **(B2)**: left multicystic dysplastic kidney of fetus case 2; **(C1)**: Horizontal gray ultrasound image of both kidneys demonstrates smaller kidney sizes with 1.0 cm of fetus case 3; **(C2)**: bilateral hyperechogenic kidneys of fetus case 3.

The mother of the first case was a 21-year-old woman, gravida one and nullipara. During prenatal follow-up at 23+3 weeks of gestation, the fetus was diagnosed to have a left multicystic dysplastic kidney and a right hyperechogenic kidney, as well as an expanded posterior corner of the lateral ventricle (10 mm in breadth). She was referred to the Prenatal Diagnosis Center of Lianyungang Maternal and Child Health Hospital (Figure 1A). After adequate genetic counseling, the couple refused amniocentesis for QF-PCR and CMA but asked for Trio-WES at 23+4 weeks. Prenatal Trio-WES results revealed that the fetus had a *de novo* deletion at 17q12 (chr17: g.34686312_36349404) and both parents tested negative for this deletion. This CNV spans 1.66 Mb, encompassing 22 OMIM genes, and was determined to be pathogenic after further analysis. Renal function and renal sonograms of the parents were both normal. After detailed genetic counseling, the family ultimately decided to terminate the pregnancy.

### Case 2

**FIGURE 2:**
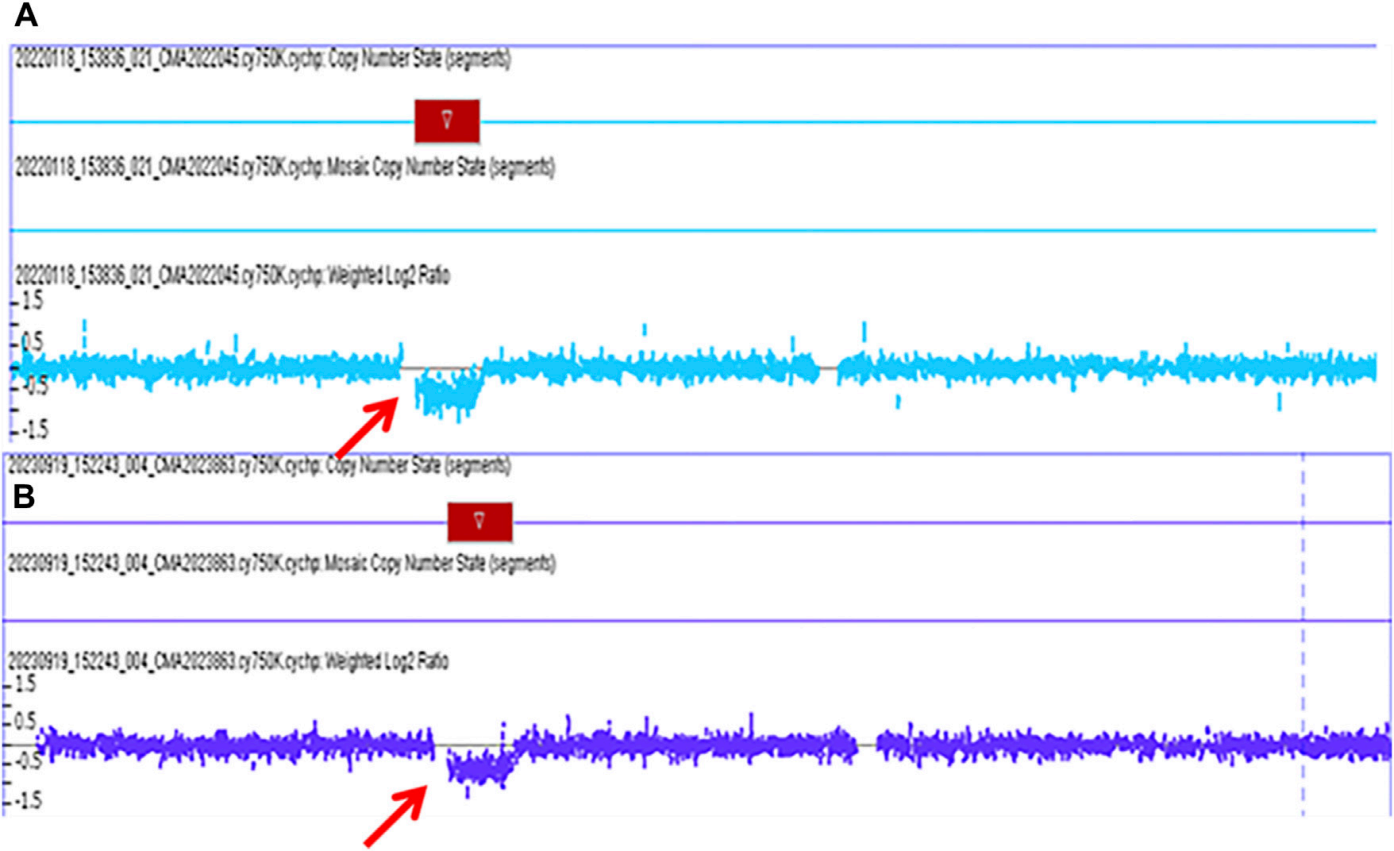
The pathogenic chromosomal microarray analysis (CMA) results of fetus case 2 and fetus case 3. **(A)**: the CMA result of case 2 revealed a 1.529 Mb deletion spanning genomic position 34,822,465-36351919 [GRCh37] in the chromosome 17q12 region. **(B)**: the CMA result of case 3 revealed a 1.494 Mb deletion spanning genomic position 34,822,465-36316144 [GRCh37] in the chromosome 17q12 region.

**FIGURE 3:**
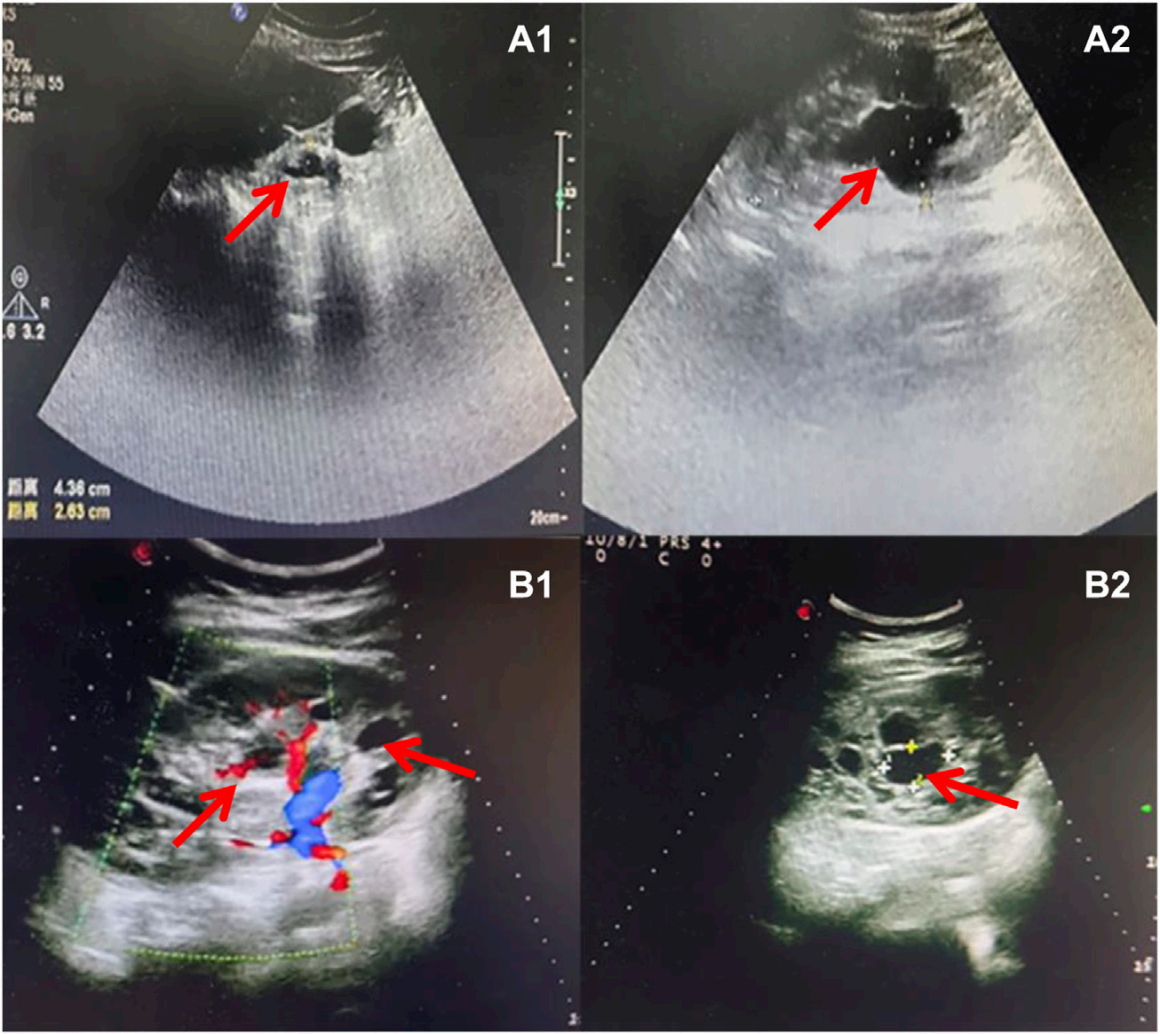
Sonographic characteristics of renal anomalies of the father (case 2) and mother (case 3) of affected fetuses. Sonographic images indicated that the father of case 2 presented with bilateral multiple renal cysts **(A1)**, with the biggest kidney cyst measuring 2.9 cm by 2.6 cm **(A2)**. Sonographic images revealed that the mother of case 3 had right renal atrophy with renal cyst **(B1)** and left hydronephrosis **(B2)**.

The pregnant mother of the second case was a 30-year-old woman, gravida one and nullipara, who was referred to the Prenatal Diagnosis Center of Lianyungang Maternal and Child Health Hospital for the left multicystic dysplastic kidney of the fetus at 24 weeks (Figure 1B1,B2). Amniocentesis for QF-PCR and CMA were performed at 24+4 weeks. No abnormality was detected by QF-PCR for chromosomes 13, 18, 21, X, and Y in this fetus. Amniocentesis for CMA revealed a 1.529 Mb deletion at 17q12, encompassing 17 OMIM genes (Figure 2A). After further analysis was performed with in-house databases and public CNV databases, including DGV, OMIM, DECIPHER, ClinGen, and ClinVar as well as reviews of literature from PubMed, the detected CNV was determined to be pathogenic. After adequate genetic counseling, the family underwent a genetic evaluation. The father was found to harbor the same deletion, and his kidney ultrasonography demonstrated bilateral polycystic kidneys with normal renal function (Figure 3A1,A2; Table 2). Even though the father with the same deletion had no other abnormal phenotypes, the parents elected to end the pregnancy.

**TABLE 2:** Clinical expression of the affected parents.

	age	Testing results		Origin	Ultrasonic features of urinary system	Renal function	Extrarenal phenotype
		SNY array	Whole exome sequencing				
Fetus 2’s father	29	arr[GRCh37]17q12(34,822,465_36410720)x1, 1.529 Mb	—	*De novo*	Bilateral multiple renal cysts	Normal	No
Fetus 3’s mother	25	—	arr[hg19]17q12(34,803,293-36104986) x1, 1.30 Mb	Unknown	Right renal atrophy and left hydronephrosis	Normal	Congenital high myopia, mild to moderate neurocognitive impairment, language development delay, maturity-onset diabetes of the young

### Case 3

The third mother was a 25-year-old woman, gravida one and nullipara, who was referred for genetic counseling at 18 weeks of gestation owing to her mental delay. Besides, speech and language delay, intellectual disability, abnormal facial features, very high myopia (more than 2000° in both eyes), abnormal posture and pace, and high blood sugar were noticed in the pregnant woman’s medical history. Then, amniocentesis for QF-PCR, CMA, and Trio-WES were performed at 18+4 weeks for diagnostic testing. QF-PCR revealed no abnormality for chromosomes 13, 18, 21, X, and Y. However, a 1.49 Mb deletion encompassing 17 OMIM genes was found at 17q12 during amniocentesis for CMA (Figure 2B). Prenatal Trio-WES results revealed that the fetus and the mother both had the deletion at 17q12 (chr17: g.34803293_36104986). This CNV was finally determined to be pathogenic after further analysis. Fetal ultrasound examination at 22+3 weeks of gestation revealed that both fetal kidneys were small, and the amount of amniotic fluid was normal (Figure 1C1,C2). While the mother’s renal ultrasonography showed right renal atrophy and severe left hydronephrosis, her renal function parameters were normal (Figure 3B,1B2; Table 2). Her ocular ultrasound examination showed high myopia fundus, axial length (>26 mm), posterior retinal process, and vitreous opacity. After detailed genetic counseling, the family decided to terminate the pregnancy. After admission, the mother’s fasting plasma glucose (FPG) and postprandial blood glucose (PBG) levels fluctuated around 5.7–6.3 mmol/L and 6.5–9.3 mmol/L, respectively. The FPG was 5.7 mmol/L (normal reference range 3.9–5.1 mmol/L) in early pregnancy. As she grew up on welfare, so we were unable to get more information about her family.

### Clinical follow-up assessment

Family 1: As the CNV was *de novo*, the couple used the natural conception for the current pregnancy. The prenatal ultrasound examination was normal without obvious abnormalities at present. Despite that, amniocentesis will be performed at the appropriate time soon to prevent gonad chimerism.

Family 2: Preimplantation genetic testing (PGT) was chosen by the couple because the CNV is inherited from the father. At 18+2 weeks of gestation, amniocentesis was performed while the karyotyping and CMA results were both negative. Furthermore, the fetal ultrasound examination was normal, and no anomalies were detected.

Family 3: The family has decided not to conceive naturally and is prepared to adopt instead.

## Discussion

In the present study, we identified three fetuses with 17q12 microdeletion prenatally. The region of chromosome 17 is highly susceptible to genomic rearrangement as it is flanked by non-allelic homologous recombination between flanking segmental duplications (Mefford et al., 2007). As a result, all patients with this microdeletion exhibit almost the same unique genetic variation of 1.4 Mb deletion, encompassing *HNF1B* plus 14 additional genes (Roehlen et al., 2018). Patients with 17q12 deletions experience a wide range of renal and extra-renal manifestations due to this disease’s heterogeneity, and its clinical manifestations mainly involve congenital abnormalities and dysfunctions in the urinary and genital systems, maturity-onset diabetes of the young type 5, and neurodevelopmental issues (Verscaj et al., 2024).

The detection rates of pathogenic CNVs in fetuses with urinary system anomalies in our study was 8.7% (4/46), which is a slightly higher rate than two Chinese studies of small cohort sizes (6.31% and 7.6%, respectively) (Hu et al., 2019; Lin et al., 2019) and a previous Chinese study with a large cohort size (8.32%, 73/877) (Su et al., 2022). Consistent with previous studies, the only phenotype detected prenatally in the current study of 17q12 deletion is renal, all coupled with *HNF1B* whole-gene deletion detected by CMA and/or Trio-WES (Moreno-De-Luca et al., 2010; Su et al., 2022). Although the most common phenotype detected prenatally for the 17q12 deletion involves renal abnormalities, its renal clinical characteristics are varied, ranging from fetal structural abnormalities like renal agenesis to ultrasonographic soft markers like fetal pyelectasis (Su et al., 2022). The *HNF1B* gene is located on chromosome 17q12, and its haploinsufficiency, including intragenic mutations and whole gene deletion, is presumably responsible for renal abnormalities in human disorders and related mouse models (Bellanné-Chantelot et al., 2004; Warren et al., 2022). About half of the affected human individuals have *HNF1B* whole-gene deletions, with point mutations found in the remaining cases (Bellanné-Chantelot et al., 2005). Human kidney development begins around the fifth week of gestation and *HNF1B* is recruited to regulate important kidney factors during urogenital development, including factors expressed in the ureteric bud and Wolffian duct epithelium to start nephrogenesis and gonad genesis (Hiesberger et al., 2004; Lokmane et al., 2010).

It is reported that approximately 70% of 17q12 deletions occur *de novo* and about 30% are inherited from parents (Mitchel et al., 2016). In the present study, the clinical phenotypes of the affected parents differed greatly in the two inherent cases (case 2 and case 3). In case 2, the affected father had only bilateral multiple renal cysts. In case 3, the mother manifested classic symptoms of 17q12 deletion syndrome as previously described (Mitchel et al., 2016), including mild to moderate neurocognitive impairment, language development delay, maturity-onset diabetes of the young (MODY), and abnormalities of the kidneys. Previous animal studies have shown that *HNF1B* is involved in hindbrain development in both zebrafish (Choe et al., 2008) and vertebrates (Pouilhe et al., 2007). Clissold et al. reported that patients with *HNFIB* deletion had higher levels of impact on psychopathology than those with intragenic mutations of *HNF1B* (Clissold et al., 2016). There are also limited reports of learning difficulties and epilepsy in patients with *HNF1B* mutations (Bingham et al., 2001; Bellanné-Chantelot et al., 2005). Besides, almost half of MODY patients have the *HNF1B* whole gene deletion (Laffargue et al., 2015). Recently, the *LHX1* gene, also known as *Lim1*, was also found to be a major and dependent regulator of renal and urogenital development and is required for epithelial tubular genesis and podocyte development in the kidney (Dormoy et al., 2011). Moreover, because LHX1 may control the development of the anterior mesendoderm, node, and midline cells, which are in charge of establishing the left-right body axis and head formation, LHX1 mutations are also believed to be linked to psychological issues and learning challenges (Costello et al., 2015). For the unreported very high myopia phenotype of the mother in case 3, no gene has been identified, although linkage has been reported to regions on the long arm of chromosomes 17 (Paluru et al., 2003; Farbrother et al., 2004).

Our study further demonstrates the wide heterogeneity of both phenotype and genotype among prenatal and adult patients related to the 17q12 deletion syndrome. The reasons for the heterogenous phenotype remain unclear. A study by Clissold et al. identified several genes that are differentially methylated in *HNF1B*-associated disease, some of which are specific to 17q12 deletion, and this may partially explain the heterogeneity (Clissold et al., 2018). Recent studies demonstrated that there may be additional gene and/or environmental modifiers that influence the *HNF1B* function (Dinneen et al., 2022). Further study is needed to explore the precise mechanism.

Notably, these fetuses in our study were all aborted although the counselors did not recommend it as this disease is not a life-threatening condition. However, due to the limitations of prenatal examinations as well as the wide heterogeneity and incomplete penetrance of 17q12 deletion syndrome, fetal phenotypes are often only partially detectable, and structural compromise is prioritized over functional compromise. These fetuses after birth may develop neurodevelopmental disorders or other extra-renal abnormalities. Due to the high sensitivity to genetic diseases by many parents in some countries including China, many families would choose to avoid the birth of an unhealthy baby through termination of the pregnancy. Reasonable and scientific evidence-based genetic counseling and a better understanding of genetic abnormalities will lead to proper management of prenatal diagnosed genetic conditions.

The current study has several limitations. First, it is based on a small sample size and the entire spectrum of neurological abnormalities related to 17q12 microdeletion remains to be fully explored in large-scale studies. Second, these fetuses all underwent induced abortions, and we were unable to assess the neurological development and the presence of other extra-renal abnormalities in their later life.

In conclusion, our findings further expand the prenatal and adult manifestations of 17q12 deletion syndrome. Additionally, genetic testing for 17q12/*HNF1B* microdeletion should be offered for a wide range of prenatally detected renal anomalies, including structural abnormality and ultrasonographic soft markers of the kidney. More importantly, our findings may allow timely supportive genetic counseling and guidance for the next pregnancy to potentially reduce the number of affected live births.

## Data Availability

The data presented in the study are deposited into CNGB Sequence Archive (CNSA) of China National GeneBank DataBase (CNGBdb) with accession number CNP0005754.
